# Supplementary material for: Nature-Inspired Nanoparticles as Paclitaxel Targeted Carrier for the Treatment of HER2-Positive Breast Cancer
Source: Cancers (Basel). 2021 May 21;13(11):2526. doi: 10.3390/cancers13112526 (PMC8196773; doi:10.3390/cancers13112526)
Supplement: Supplementary file 1 [file cancers-13-02526-s001.zip › cancers-1201324-supplementary.pdf]

Nature-inspired nanoparticles as paclitaxel targeted carrier for the treatment of HER2-positive breast cancer

Celia Nieto*, Milena A. Vega and Eva Martín del Valle*

Supplementary Material

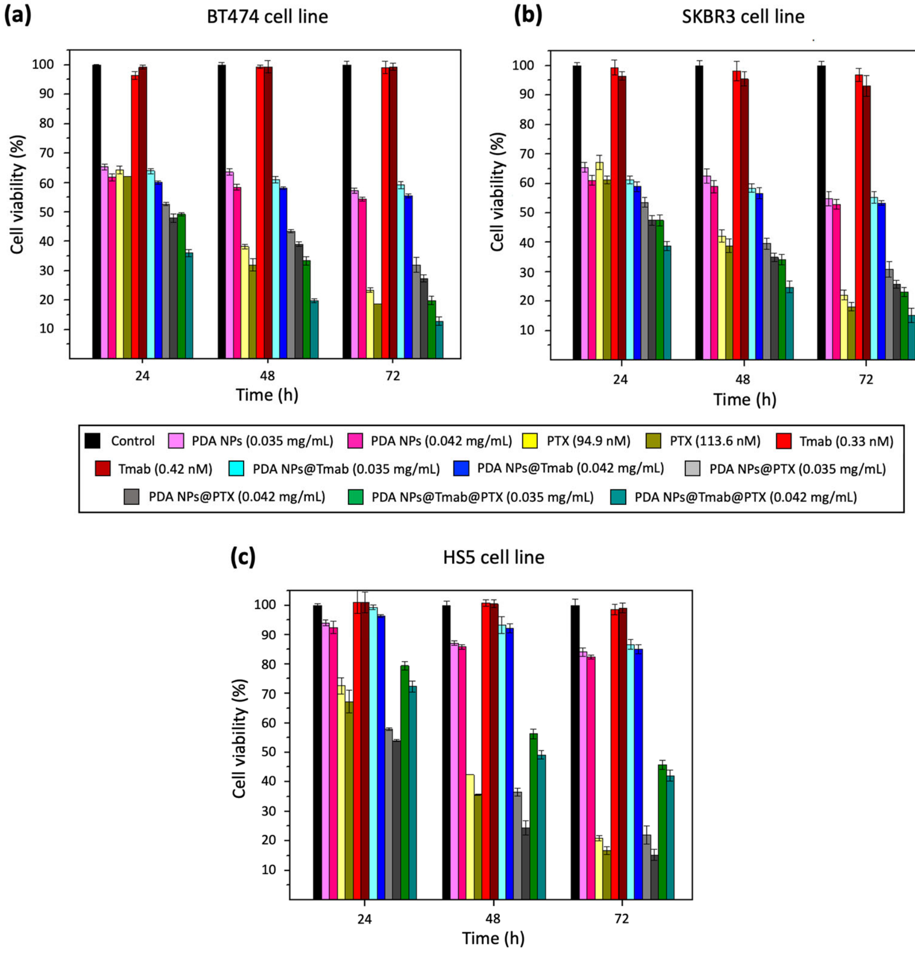

Figure S1. Survival rates of BT474 **(a)**, SKBR3 **(b)** and HS5 **(c)** cells after treatment with 0.035 and 0.042 mg/mL bare PDA NPs, PDA NPs@Tmab, PDA NPs@PTX and PDA NPs@Tmab@PTX, as well as with concentrations of free Tmab and PTX similar to those adsorbed in PDA NPs.

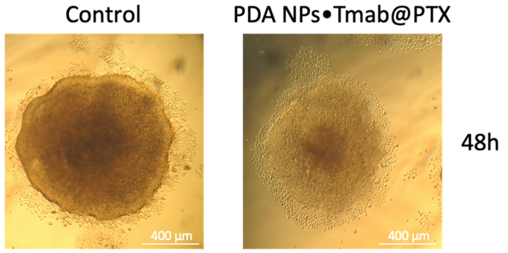

Figure S2. Phase-contrast images of BT474 spheroids 48 hours after treatment with or without PDA NPs•Tmab@PTX (0.035 mg/mL).

Table S1. Surface charge of the PDA NPs synthesized using 2-PrOH before and after loading them with PTX (through adsorption) and Tmab (through adsorption and the carbodiimide chemistry).

	Zeta potential (mV)
**PDA NPs**	-27.4 ± 1.5
**PDA NPs@Tmab@PTX**	-25.8 ± 1.3
**PDA NPs•Tmab@PTX**	-24.3 ± 1.5

Table S2. T- and p-values obtained when an statical analysis was performed in order to determine if there were significant differences between the toxicity of the different loaded PDA NPs and equivalent concentrations of PTX to the BT474 cell line. It was considered that p-values inferior to 0.05 were statistically significant.

	**BT474 cell line**		
**Treatment condition**	**Time (hours)**	**t-value**	**p-value**
PTX (99.5 nM) *vs.* PDA NPs@Tmab@PTX (0.035 mg/mL)	24	6.707	**0.0025**
	48	11.789	**0.0003**
	72	3.112	**0.0358**
PTX (119.5 nM) *vs.* PDA NPs@Tmab@PTX (0.042 mg/mL)	24	19.827	**3.8171 E-05**
	48	25.836	**1.3332 E-05**
	72	8.438	**0.0011**
PTX (99.5 nM) *vs.* PDA NPs•Tmab@PTX (0.035 mg/mL)	24	12.615	**0.0002**
	48	4.620	**0.0098**
	72	6.241	**0.0034**
PTX (119.5 nM) *vs.* PDA NPs•Tmab@PTX (0.042 mg/mL)	24	35.561	**3.7323 E-06**
	48	12.168	**0.0003**
	72	10.515	**0.0005**

Table S3. T- and p-values obtained when an statical analysis was performed in order to determine if there were significant differences between the toxicity of the different loaded PDA NPs and equivalent concentrations of PTX to the SKBR3 cell line. It was considered that p-values inferior to 0.05 were statistically significant.

	**SKBR3 cell line**		
**Treatment condition**	**Time (hours)**	**t-value**	**p-value**
PTX (99.5 nM) *vs.* PDA NPs@Tmab@PTX (0.035 mg/mL)	24	12.028	**0.0003**
	48	10.525	**0.0005**
	72	20.210	**3.5388 E-05**
PTX (119.5 nM) *vs.* PDA NPs@Tmab@PTX (0.042 mg/mL)	24	8.038	**0.0013**
	48	8.466	**0.0011**
	72	8.614	**0.0010**
PTX (99.5 nM) vs. PDA NPs•Tmab@PTX (0.035 mg/mL)	24	8.187	**0.0012**
	48	5.943	**0.0040**
	72	0.412	**0.7011**
PTX (119.5 nM) *vs.* PDA NPs•Tmab@PTX (0.042 mg/mL)	24	14.798	**0.0001**
	48	3.280	**0.0304**
	72	3.699	**0.0209**

Table S4. T- and p-values obtained when an statical analysis was performed in order to determine if there were significant differences between the toxicity of the different loaded PDA NPs and equivalent concentrations of PTX to the HS5 cell line. It was considered that p-values inferior to 0.05 were statistically significant.

	**HS5 cell line**		
**Treatment condition**	**Time (hours)**	**t-value**	**p-value**
PTX (99.5 nM) vs. PDA NPs@Tmab@PTX (0.035 mg/mL)	24	2.145	**0.0985**
	48	9.238	**0.0008**
	72	7.684	**0.0015**
PTX (119.5 nM) vs. PDA NPs@Tmab@PTX (0.042 mg/mL)	24	0.890	**0.4238**
	48	9.701	**0.0006**
	72	33.161	**4.9319 E-06**
PTX (99.5 nM) vs. PDA NPs•Tmab@PTX (0.035 mg/mL)	24	5.262	**0.0062**
	48	80.584	**1.4213 E-07**
	72	88.264	**9.8774 E-08**
PTX (119.5 nM) vs. PDA NPs•Tmab@PTX (0.042 mg/mL)	24	23.727	**1.8710 E-05**
	48	22.825	**2.1824 E-05**
	72	43.853	**1.6168 E-06**

Table S5. T- and p-values obtained when an statical analysis was performed in order to determine if there were significant differences between the toxicity of PDA NPs@Tmab@PTX and PDA NPs•Tmab@PTX to the BT474 cell line. It was considered that p-values superior to 0.05 were not statistically significant.

	BT474 cell line		
Treatment condition	Time (hours)	t-value	p-value
PDA NPs@Tmab@PTX vs. PDA NPs•Tmab@PTX (0.035 mg/mL)	24	2.501	**0.0667**
	48	6.065	**0.0037**
	72	4.613	**0.0099**
PDA NPs@Tmab@PTX vs. PDA NPs•Tmab@PTX (0.042 mg/mL)	24	10.741	**0.0004**
	48	1.475	**0.2142**
	72	2.994	**0.0402**

Table S6. T- and p-values obtained when an statical analysis was performed in order to determine if there were significant differences between the toxicity of PDA NPs@Tmab@PTX and PDA NPs•Tmab@PTX to the SKBR3 cell line. It was considered that p-values superior to 0.05 were not statistically significant.

	SKBR3 cell line		
Treatment condition	Time (hours)	t-value	p-value
PDA NPs@Tmab@PTX vs. PDA NPs•Tmab@PTX (0.035 mg/mL)	24	6.313	**0.0032**
	48	2.115	**0.1019**
	72	0.402	**0.7080**
PDA NPs@Tmab@PTX vs. PDA NPs•Tmab@PTX (0.042 mg/mL)	24	4.926	**0.0079**
	48	0.346	**0.7468**
	72	1.282	**0.1425**

Table S7. T- and p-values obtained when an statical analysis was performed in order to determine if there were significant differences between the toxicity of PDA NPs@Tmab@PTX and PDA NPs•Tmab@PTX to the HS5 cell line. It was considered that p-values superior to 0.05 were not statistically significant.

	HS5 cell line		
Treatment condition	Time (hours)	t-value	p-value
PDA NPs@Tmab@PTX vs. PDA NPs•Tmab@PTX (0.035 mg/mL)	24	0.088	**0.9341**
	48	3.970	**0.0165**
	72	1.694	**0.1654**
PDA NPs@Tmab@PTX vs. PDA NPs•Tmab@PTX (0.042 mg/mL)	24	0.395	**0.7133**
	48	9.790	**0.0006**
	72	2.671	**0.0557**
